# Influences of age-related changes in mesenchymal stem cells on macrophages during in-vitro culture

**DOI:** 10.1186/s13287-017-0608-0

**Published:** 2017-06-24

**Authors:** Yuan Yin, Rui-Xin Wu, Xiao-Tao He, Xin-Yue Xu, Jia Wang, Fa-Ming Chen

**Affiliations:** State Key Laboratory of Military Stomatology and National Clinical Research Center for Oral Diseases, Department of Periodontology, School of Stomatology, Fourth Military Medical University, 145th West Changle Road, Xi’an, 710032 People’s Republic of China

**Keywords:** Cell immunomodulation, Macrophage polarization, Cell aging, Cell–cell interaction, Cellular therapy

## Abstract

**Background:**

Mesenchymal stem cells (MSCs) have been widely used in cytotherapy and tissue engineering due to their immunosuppressive ability and regenerative potential. Recently, the immunomodulatory influence of MSCs has been gaining increasing attention because their functional roles in modulating immune responses likely have high clinical significance.

**Methods:**

In this study, we investigated the influence of MSCs on macrophages (Mφs) in in-vitro cell culture systems. Given evidence that aged MSCs are functionally compromised, bone marrow-derived MSCs (BMSCs) isolated from both young and aged mice (YMSCs and AMSCs) were evaluated and contrasted.

**Results:**

We found that YMSCs exhibited greater proliferative and osteo-differentiation potential compared to AMSCs. When cocultured with RAW264.7 cells (an Mφ cell line), both YMSCs and AMSCs coaxed polarization of Mφs toward an M2 phenotype and induced secretion of anti-inflammatory and immunomodulatory cytokines. Compared to AMSCs, YMSCs exhibited a more potent immunomodulatory effect. While Mφs cocultured with either YMSCs or AMSCs displayed similar phagocytic ability, AMSC coculture was found to enhance Mφ migration in Transwell systems. When BMSCs were prestimulated with interferon gamma before coculture with RAW264.7 cells, their regulatory effects on Mφs appeared to be modified. Here, compared to stimulated AMSCs, stimulated YMSCs also exhibited enhanced cellular influence on cocultured RAW264.7 cells.

**Conclusions:**

Our data suggest that BMSCs exert an age-related regulatory effect on Mφs with respect to their phenotype and functions but an optimized stimulation to enhance MSC immunomodulation is in need of further investigation.

## Background

Age-related changes in mesenchymal stem cell (MSC) properties have attracted considerable attention in the field of tissue engineering and regenerative medicine. It is now well recognized that aging MSCs exhibit compromised potential toward proliferation, target migration, tissue-specific differentiation and downstream tissue regeneration and angiogenesis [1–4]. Fortunately, evidence showing that cell pretreatments with hypoxia, growth factors, conditioned medium and other conditions during in-vitro culture may rescue, at least to a certain degree, the lost functionalities of stem cells due to aging or long-term ex-vivo cell expansion, which promotes functional outcomes following in-vivo cell transplantation [5–10]. To achieve a successful regenerative outcome, a growing corpus of studies have recently highlighted the immunomodulatory roles of therapeutic MSCs because they act as key contributors during the wound healing cascade largely by modulating immunological responses, instead of or in addition to participating in building the new tissue [4, 11–14]. In this context, MSCs create an immunomodulatory microenvironment that minimizes continued tissue damage due to inflammation and facilitates reparative and regenerative processes through regulation of immune cell populations (reviewed in [15]).

Along with advances in the understanding of immune responses during tissue repair, the functional role of MSCs in tissue repair has undergone considerable evolution [16]. Macrophages (Mφs) are major players in the progression of inflammation as well as tissue repair via classic (M1) or alternative (M2) polarization [17]. The two distinct states of Mφs elicit divergent effects: the M1 phenotype cells upregulate activities related to tissue destruction via secretion of numerous proinflammatory cytokines, such as tumor necrosis factor alpha (TNFα), interleukin-1β (IL-1β), interleukin-12 (IL-12) and interleukin-6 (IL-6); whereas cells with an M2 subtype support tissue remodeling by releasing large amounts of anti-inflammatory cytokines, such as interleukin-10 (IL-10) and transforming growth factor beta (TGFβ) [18, 19]. Therefore, Mφs can promote both positive and negative outcomes depending on context-dependent polarization profiles, and transition from an M1 phenotype to an M2 phenotype is a key strategy to control and promote tissue regeneration [20, 21]. Although concerted efforts have been placed into using biomaterials to coax Mφ polarization, investigations into interactions between MSCs and Mφs also substantiate the fact that functional MSCs can drive protective M2 polarization [22–27]. The immunomodulatory effect of MSCs on Mφs offers a new concept for facilitating tissue regeneration and cannot be ignored in materials-based tissue engineering designs [28–31]. However, the impacts of aging on the immunomodulatory properties of MSCs, particularly their direct regulatory effect on Mφs, remain largely unknown.

In the present study, we compared the modulation abilities of MSCs isolated from young and aged mice in terms of their influence on Mφ phenotype and function. Given that interferon gamma (IFNγ) can activate and potentially enhance the immunomodulatory properties of MSCs [32–34], we in parallel investigated how IFNγ-prestimulated MSCs influence Mφs. Our data provide additional evidence for the effects of aging on stem cell properties, which may have clinical significance for future cytotherapy and tissue engineering applications.

## Methods

### Isolation of mouse BMSCs

Male C57BL/6 J mice (aged 6–8 weeks or aged over 24 weeks) were obtained from the Laboratory Animal Research Centre of the Fourth Military Medical University (FMMU, Xi’an, China). The use of animals for cell isolation was approved by the Animal Use and Care Committee of FMMU, and the experimental procedures were in accordance with the Intramural Animal Use and Care Committee of FMMU. Mouse BMSCs were isolated and cultured as described previously [35]. Briefly, mice were killed, and hind limbs were aseptically removed to obtain bones for cell isolation. Bone marrow tissue from the femur and tibia was flushed out and incubated in basal culture medium comprising α-minimum essential medium (α-MEM; HyClone, Logan, UT, USA) supplemented with 20% fetal bovine serum (FBS; Hangzhou Sijiqing Biological Engineering Materials Co., Ltd, Hangzhou, China) and 1% penicillin and streptomycin (HyClone) in 10-cm diameter wells; nonadherent cells were removed via medium change after 72 h. The remaining adherent colonies were cultured for 7–10 days until 70–80% confluence (medium was replaced every 3 days) and passaged after digestion with 0.25% trypsin (Invitrogen Life Technologies, Carlsbad, CA, USA). BMSCs obtained from animals aged 6–8 weeks were designated young BMSCs (YMSCs), while cells from animals aged over 24 weeks were designated aged BMSCs (AMSCs). Both YMSCs and AMSCs at passages 2–3 were used for the following experiments.

### Cell characterization of BMSCs

#### CCK-8 assay

Cell proliferation was quantitatively determined with a 7Sea Cell Counting Kit (7Sea Biotech, Shanghai, China). YMSCs and AMSCs were seeded in 96-well culture plates separately at a density of 3000 cells per well with 200 μl of basal culture medium. After 12 h for cell adhesion, the medium was refreshed every other day, and the CCK-8 assay was performed every day during the 7-day incubation. Each day at a proscribed time point, 20 μl of CCK-8 reagent was added to each test well, and the plates were incubated at 37 °C in the dark for 2 h. The incubated medium was then transferred to a new 96-well plate, and the absorbance was measured at 450 nm with a microplate reader (ELx800; BioTek Instruments Inc., Highland Park, FL, USA).

#### EdU incorporation assay

An EdU assay was used to evaluate cell proliferation capacities by investigating the rate of DNA synthesis in cells. YMSCs and AMSCs were seeded in a six-well plate (2 × 10^5^ cells per well) and cultured for 4 days until the cells reached 70–80% confluence before the 5-ethynyl-2′-deoxyuridine (EdU) incorporation assay, which was performed with a keyFluor488 Click-iT EdU kit (keyGEN BioTECH, Nanjing, China) according to the manufacturer’s protocol. EdU-positive cells were visualized with an inverted fluorescence microscope (Olympus, Tokyo, Japan).

#### Osteogenic differentiation assay

YMSCs and AMSCs were plated in 12-well dishes at a density of 1 × 10^5^ cells/well and cultured until the cells reached 70% confluence. The culture medium was then changed to osteogenic-inducing medium; that is, basal culture medium supplemented with 50 μg/ml vitamin C, 10 nM dexamethasone and 10 mM β-glycerophosphate (all from Sigma–Aldrich, St. Louis, MO, USA). After 14 days of osteogenic induction, cells were fixed with 4% paraformaldehyde (MP Biomedicals, Santa Ana, CA, USA) for 30 min at room temperature, and Alizarin Red S (Sigma–Aldrich) was used to stain calcium deposits, which were observed with an inverted microscope (Olympus) and photographed. The stained areas were dissolved with 100 mM cetylpyridinium chloride for 30 min at room temperature, and the OD values of the resultant solutions representing the osteogenic potential of BMSCs were measured at 560 nm with a microplate reader.

#### Adipogenic differentiation assays

To measure the adipogenic capacity of BMSCs, YMSCs and AMSCs were seeded separately in 12-well plates (1 × 10^5^ cells per well) and cultured until the cells reached 80% confluence. Subsequently, the cells were induced with adipogenic medium; that is, basal culture medium supplemented with 0.5 mM 3-isobutyl-1-methylxanthine (IBMX), 1 μM dexamethasone, 0.1 mM indomethacin and 10 μg/ml insulin (all from Sigma–Aldrich). After 7 days of adipogenic induction, cells were fixed with 4% paraformaldehyde for 30 min at room temperature, and Oil Red O (Sigma–Aldrich) was used to measure intracellular lipid droplet accumulation. Oil Red O-positive areas were observed with an inverted microscope (Olympus), photographed and then dissolved in isopropanol. OD values of the resultant solutions representing the adipogenic potential of BMSCs were quantitatively measured at 560 nm with a microplate reader.

### Coculture of macrophages with YMSCs and AMSCs

Coculture tests of RAW264.7 and BMSCs were performed with Millicell® hanging cell culture inserts (polyethylene terephthalate (PET) membranes with 0.4 μm pores) (Millipore, Billerica, MA, USA). Briefly, BMSCs were seeded in the upper compartment (inserts) and RAW264.7 cells were plated in six-well plates; each underwent a 12-h cell adherence incubation in basal culture medium, and then the inserts were placed onto the six-well plates to obtain physically separated coculture of the two types of cells (RAW264.7: BMSCs = 10:1). After coculture for 48 h, RAW264.7 cells in the lower compartment (six-well plate) were collected for analysis (named the RAW264.7 + YMSC group and the RAW264.7 + AMSC group). Given that proinflammatory cytokine-stimulated BMSCs have an enhanced immunomodulatory effect on Mφs [33], we preconditioned BMSCs with IFNγ at a concentration of 20 ng/ml, and then the IFNγ-stimulated BMSCs were used for coculture (named the RAW264.7 + stimulated YMSC group and the RAW264.7 + stimulated AMSC group). RAW264.7 cells without coculture were used as the control, and both unstimulated BMSCs and stimulated BMSCs cocultured with RAW264.7 cells were investigated in parallel.

### Phenotype of RAW264.7 cells following coculture with BMSCs

#### Flow cytometry analysis

The cell surface markers of RAW264.7 cells (before or following coculture with YMSCs and AMSCs (with or without IFNγ stimulation)) were analyzed by flow cytometry. Briefly, cells were trypsinized, washed twice with PBS (HyClone) and divided into 1 × 10^6^ cells/tube. For blocking of Fc receptors in the flow cytometric analysis, the cells were preincubated with purified anti-CD16/CD32 antibody (reached a concentration of 1%; Biolegend, San Diego, CA, USA) for 10 min on ice. Then, antibodies for cell markers were added to the indicated tubes (antibodies against mouse CD11b and F4/80 were used to stain untreated RAW264.7 cells); RAW264.7 cells (and the control cells) were stained separately with antibodies against mouse CD86 and CD206 following coculture and mixed into the final antibody concentration (antibodies against mouse CD11b, F4/80 and CD206 reached a concentration of 1%; antibodies against mouse CD86 reached a concentration of 6.25‰; all from Biolegend). Cells not treated with antibodies were defined as the blank controls. Cell suspension with antibodies was incubated on ice for 30 min in the dark and washed twice with PBS. The positively stained cells were detected with a Beckman Coulter Epics XL cytometer (Beckman Coulter, Fullerton, CA, USA).

#### Immunofluorescence staining

RAW264.7 cells were adhered to the surface of glass cover slides before coculture, and then the glass cover slides were transferred to Transwell systems. After coculture, cells on the slides were fixed with 4% paraformaldehyde, permeabilized with 0.3% Triton (MP Biomedicals) for 10 min and then blocked with 1% bovine serum albumin (BSA; MP Biomedicals) for 30 min at room temperature. Slides were incubated separately with antibodies against mouse CD206 (1/400, ab64693; Abcam, Cambridge, UK) or inducible nitric oxide synthase (iNOS) (1/400, ab178945; Abcam) at 4 °C overnight. After being washed in PBS, the slides were incubated with Alexa Fluor® 594 goat-anti-rabbit IgG secondary antibodies (1/500; Jackson ImmunoResearch Laboratories, West Grove, PA, USA) for 2 h in the dark at room temperature. Finally, slides were counterstained with PBS solution containing 4′,6-diamidino-2-phenylindole (DAPI) (Invitrogen Life Technologies) and FITC-labeled phalloidin (Yeasen Biotech Inc., Shanghai, China) for 30 min in the dark. DAPI was used to label cell nuclei, and phalloidin was used to visualize the cytoskeleton. All images were obtained using a confocal laser scanning microscope (FV1000; Olympus) and photographed with Fluoview 1000 (Olympus).

### Characteristics of RAW264.7 cells following coculture with BMSCs

#### Quantitative real-time polymerase chain reaction

Quantitative real-time polymerase chain reaction (qRT-PCR) was used to examine mRNA expression levels in RAW264.7 cells. Total RNA from RAW264.7 cells was isolated with RNAiso Plus (Takara, Tokyo, Japan), followed by cDNA synthesis using PrimeScript™ RT Master Mix (Perfect Real Time; Takara) according to the manufacturer’s instructions. qRT-PCR was performed on 5-fold-diluted cDNA samples in double-distilled water using SYBR® Premix Ex Taq™ II (Tli RNaseH Plus; Takara), and each 10 μl reaction contained 5 μl of SYBR mix, 2 μl of cDNA, 2.2 μl of ddH_2_O, 0.4 μl of forward primer and 0.4 μl of reverse primer. Mouse-specific primers for quantification of *TNFα*, *IL-1β*, *IL-10*, *TGFβ*, *Arginase-1* (*Arg1*) and *iNOS* were used to perform qRT-PCR (Table 1). Glyceraldehyde 3-phosphate dehydrogenase (*GAPDH*) was used to normalize the expression level of related genes.

**Table 1:** Primer sequences of target genes

Primer		Sequence (5′–3′)
*TNF-α*	Forward	TATGGCCCAGACCCTCACA
	Reverse	GGAGTAGACAAGGTACAACCCATC
*IL-1β*	Forward	AAGGAGAACCAAGCAACGACAAAA
	Reverse	TGGGGAACTCTGCAGACTCAAACT
*iNOS*	Forward	CAAGCTGAACTTGAGCGAGGA
	Reverse	TTTACTCAGTGCCAGAAGCTGGA
*IL-10*	Forward	GCCAGAGCCACATGCTCCTA
	Reverse	GATAAGGCTTGGCAACCCAAGTAA
*Arg1*	Forward	AGCTCTGGGAATCTGCATGG
	Reverse	ATGTACACGATGTCTTTGGCAGATA
*TGF-β*	Forward	CAAGCTGAACTTGAGCGAGGA
	Reverse	TTTACTCAGTGCCAGAAGCTGGA
*GAPDH*	Forward	CCAATGTGTCCGTCGTGGATCT
	Reverse	GTTGAAGTCGCAGGAGACAACC

*TNFα* tumor necrosis factor alpha, *IL* interleukin, *iNOS* inducible nitric oxide synthase, *Arg1* Arginase-1, *TGFβ* transforming growth factor beta, *GAPDH* glyceraldehyde 3-phosphate dehydrogenase

#### Measurement of nitric oxide production

The Griess method was applied to evaluate nitric oxide (NO) production in RAW264.7 cells. Griess reagent is based on the chemical diazotization reaction, and a nitrite detection kit (Beyotime Biotech Inc., Hangzhou, China) was used for the assay according to the manufacturer’s instructions. Briefly, supernatant from each group (after 48 h of coculture) or standard NaNO_2_ was mixed with Griess reagents serially in a 96-well plate. After a 10-min incubation, OD values of the mixtures were measured quantitatively at 540 nm with the reference filter set at 630 nm.

#### Enzyme-linked immunosorbent assay

The concentration of TNFα and IL-10 in the supernatants of RAW264.7 cells (following coculture with YMSCs or AMSCs (with or without IFNγ stimulation)) was examined with enzyme-linked immunosorbent assay (ELISA) kits (Neobioscience, Guangzhou, China). After 48 h of coculture, the supernatants of each group as well as the control (RAW264.7 cells cultured alone) were collected and assayed according to the manufacturer’s instructions.

#### Phagocytic assay

To determine the phagocytic activity of RAW264.7 cells, the cells (following coculture with YMSCs and AMSCs (with or without IFNγ stimulation)) were incubated with chicken red blood cells (CRBCs; Cellbio, Shanghai, China) and then examined with eosin-methylene blue staining (Romanowsky staining), fluorescence staining and flow cytometry. For Romanowsky staining, CRBCs (2 × 10^6^ cells per well) were suspended in fresh basal culture medium and the suspension replaced the culture medium of RAW264.7 cells, and cells were then incubated at 37 °C for 20 min; ultimately, all cells were fixed with 4% paraformaldehyde and stained with eosin-methylene blue dye (Solarbio, Beijing, China) according to the instructions. For fluorescence staining and flow cytometry, CRBCs were prelabeled with 1:500 PKH-26 dye (Sigma–Aldrich). After being washed and resuspended in basal culture medium, the suspension of CRBCs (2 × 10^6^ cells per well) replaced the culture medium of RAW264.7 cells (following coculture with YMSCs and AMSCs (with or without IFNγ stimulation)), and the cell mixture was incubated at 37 °C for 20 min. Ultimately, all cells were fixed and subjected to Hoechst staining as described previously in this manuscript. With regard to flow cytometry, cells were directly trypsinized and exposed to FITC-F4/80 (Biolegend) antibody as described previously in this manuscript.

#### Migratory ability assay

The migratory ability of RAW64.7 cells was determined using migration Transwell systems. The migration Transwell system contained 8-μm-pore Millicell® hanging cell culture inserts (Millipore) in 24-well culture plates. There were two types of migration assay: first YMSCs, AMSCs, stimulated YMSCs and stimulated AMSCs were incubated separately in 24-well plates for 12 h (2 × 10^4^ cells per well). Then, RAW264.7 cells (untreated) were digested and transferred into the upper inserts of plates (2 × 10^5^ cells per well) with preincubated BMSCs in the lower chambers (named the BMSC chemotaxis migration assay). The system was incubated for 10 h, and the cells on the membranes of the Transwell inserts were fixed with 4% paraformaldehyde. After detaching nonmigrated cells on the upper surface of inserts, cells that migrated to the lower surface were stained with 0.3% crystal violet (Sigma–Aldrich) for 30 min. Cells in five random fields were counted for statistical analysis. On the other hand, RAW264.7 cells following coculture with YMSCs and AMSCs (with or without IFNγ stimulation) were digested and transferred to the upper chamber of the Transwell inserts (named the Mφ migration assay). The system was incubated for 10 h, and the inserts were fixed, cleaned and stained as described previously.

### Statistical analysis

All values are presented as the mean ± standard deviation (SD) from at least three independent experiments. Comparisons between groups were analyzed with GraphPad Prism 5 software using one-way analysis of variance (ANOVA) followed by Tukey’s post-hoc test. *p* < 0.05 was considered to indicate a significant difference.

## Results

### Characterization of YMSCs and AMSCs

**Fig. 1:**
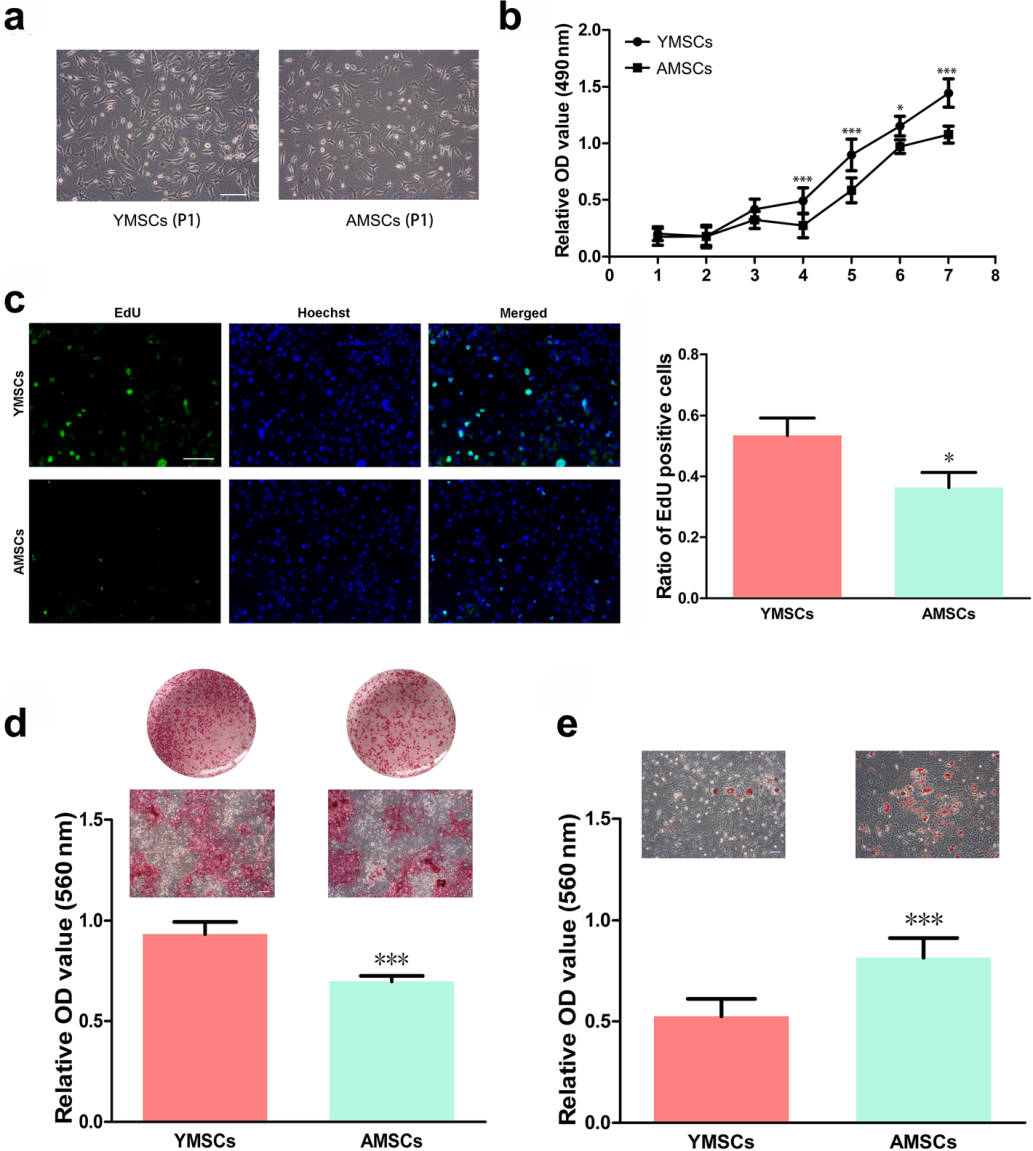
Age-related differences in morphology, cell proliferation and osteogenic/adipogenic differentiation of mouse BMSCs. **a** Images of BMSCs showing cell morphology at passage 1 (*scale bar*: 100 μm). **b** Growth curves of YMSCs and AMSCs (determined by CCK-8 assays). **c** Cell viability (EdU assays) of YMSCs and AMSCs, and statistical analysis (performed by comparing the number of EdU-positive cells in five random fields). **d** Potential of mouse YMSCs and AMSCs toward osteogenic differentiation analyzed via Alizarin Red S staining; images of Alizarin Red S-stained mineral nodules were acquired with an inverted microscope after 14 days of osteogenic induction (*scale bar*: 100 μm) and data analysis. **e** Potential of mouse YMSCs and AMSCs toward adipogenic differentiation analyzed via Oil Red O staining; images of Oil Red O-stained intracellular lipid droplets were acquired with an inverted microscope after 7 days of adipogenic induction (*scale bar*: 100 μm) and data analysis. Data presented as mean ± standard deviation. *p < 0.05, ***p < 0.001, a significant difference between YMSCs and AMSCs at the same time point (**b**) or between the displayed columns (**c**–**e**). *AMSC* aged bone marrow-derived mesenchymal stem cell, *YMSC* young bone marrow-derived mesenchymal stem cell, *P1* passage 1, *OD* optical density, *EdU* 5-ethynyl-2′-deoxyuridine

Both YMSCs and AMSCs exhibited a fibroblastic spindle morphology (Fig. 1a). In terms of CCK-8 assays, YMSCs showed higher proliferative rates than AMSCs from day 4 to day 7 (*p* < 0.05 or *p* < 0.001) (Fig. 1b). When the EdU assay was additionally assessed at day 4, the ratio of EdU-positive cells was significantly higher in YMSCs than in AMSCs (*p* < 0.001) (Fig. 1c). Although both YMSCs and AMSCs were capable of osteogenic differentiation, the mineral nodules generated by YMSCs were greater in number and larger in size when observed with an inverted microscope, and this difference was further demonstrated by quantitative analysis of calcium deposits (*p* < 0.001) (Fig. 1d). For cell adipogenic differentiation, AMSCs produced more intracellular lipid droplets than YMSCs (*p* < 0.001) (Fig. 1e).

### Cell surface markers of RAW264.7 cells following coculture

**Fig. 2:**
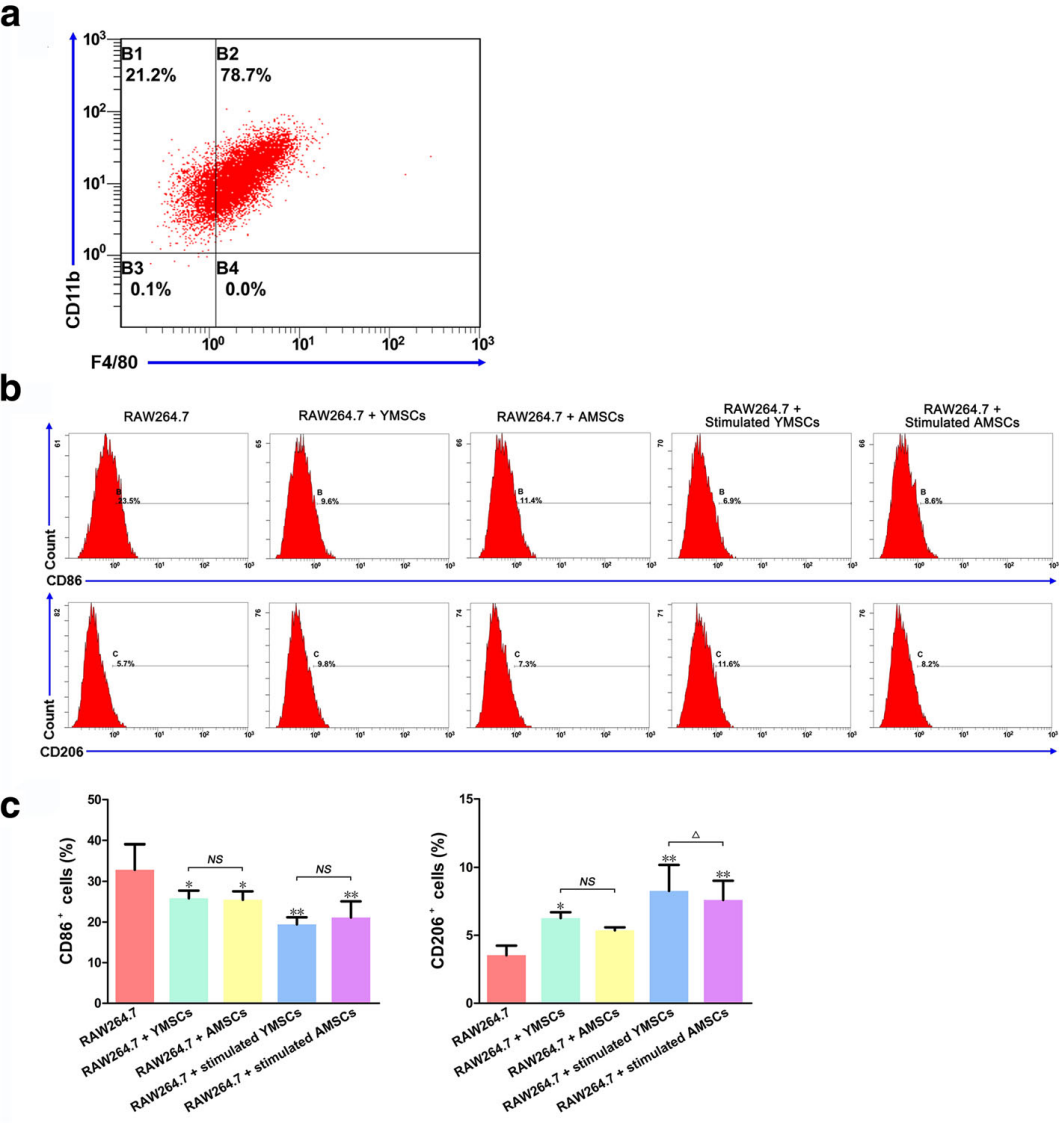
Cell surface markers (analyzed via flow cytometry) in RAW264.7 cells before or following coculture with BMSCs. **a** Markers CD11b and F4/80 in RAW264.7 cells before coculture. **b** Markers for M1 Mφs (CD86) and M2 Mφs (CD206) in RAW264.7 cells following coculture with YMSCs and AMSCs (with or without IFNγ stimulation); RAW264.7 cells without coculture served as control. **c** Data analysis of cell surface markers (CD86 and CD206) following coculture. Data presented as mean ± standard deviation. *p < 0.05, **p < 0.01, a significant difference when compared to control; ^△^ *p* < 0.05, a significant difference between the indicated groups; ^*NS*^ *p* > 0.05, no significant difference between the indicated groups. *AMSC* aged bone marrow-derived mesenchymal stem cell, *YMSC* young bone marrow-derived mesenchymal stem cell

**Fig. 3:**
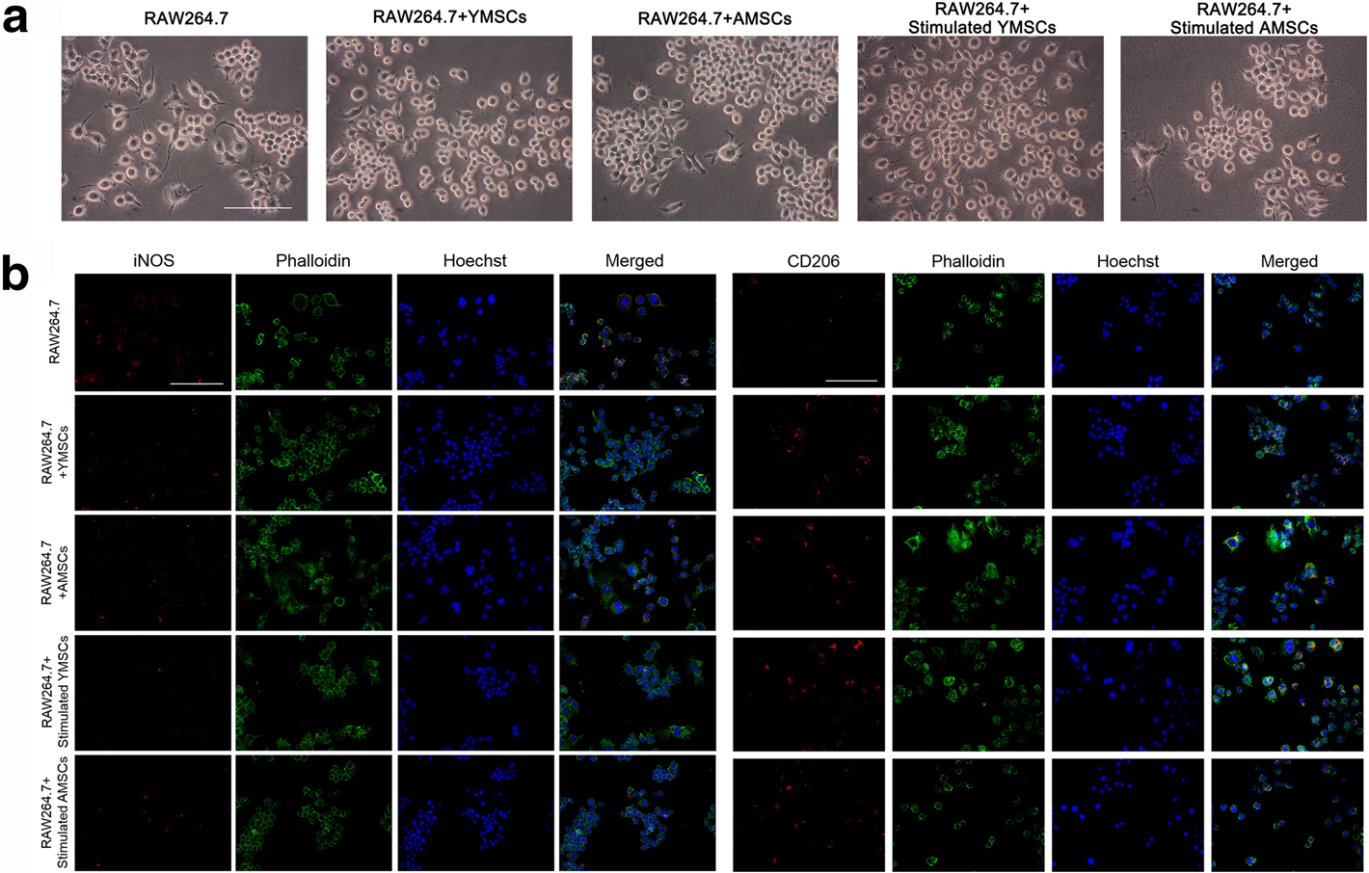
Cell morphology (**a**) and immunofluorescence (**b**) of RAW264.7 cells following coculture with YMSCs and AMSCs (with or without IFNγ stimulation); RAW264.7 cells without coculture served as control. **a** Images of RAW264.7 cells in different groups were obtained with an inverted microscope (*scale bar*: 100 μm). **b** Specific markers for the proinflammatory phenotype (iNOS) and anti-inflammatory phenotype (CD206) were stained via immunofluorescence (iNOS and CD206 were labeled with Alexa Fluor® 594 secondary antibodies, cytoskeleton was labeled with FITC-phalloidin, cell nucleus was labeled with Hoechst; *scale bar*: 100 μm). *AMSC* aged bone marrow-derived mesenchymal stem cell, *YMSC* young bone marrow-derived mesenchymal stem cell, *iNOS* inducible nitric oxide synthase

When RAW264.7 cells (before coculture) were subjected to a flow cytometry assay, the double-positive ratio of F4/80 (specific for Mφs) and CD11b (specific myeloid markers) was 78.7% (Fig. 2a). Following coculture with either IFNγ-stimulated or unstimulated BMSCs, CD86 expression in RAW264.7 cells significantly decreased while CD206 expression increased when compared to the control (RAW264.7 cells without coculture) (*p* < 0.01 or *p* < 0.05) (Fig. 2b, c). Except for stimulated YMSCs, which led to a higher level of CD206 in cocultured RAW264.7 cells compared to cells cocultured with stimulated AMSCs, there was no significant difference in surface marker expression among the other matched groups (*p* > 0.05) (Fig. 2b, c). RAW264.7 cells exhibited a relatively small, round and regular shape following coculture with BMSCs (Fig. 3a). With respect to the immunofluorescence assay, coculture with either YMSCs or AMSCs, with or without stimulation, consistently promoted CD206 expression in RAW264.7 Mφs while iNOS expression was decreased (Fig. 3b).

### mRNA expression of RAW264.7 cells following coculture

**Fig. 4:**
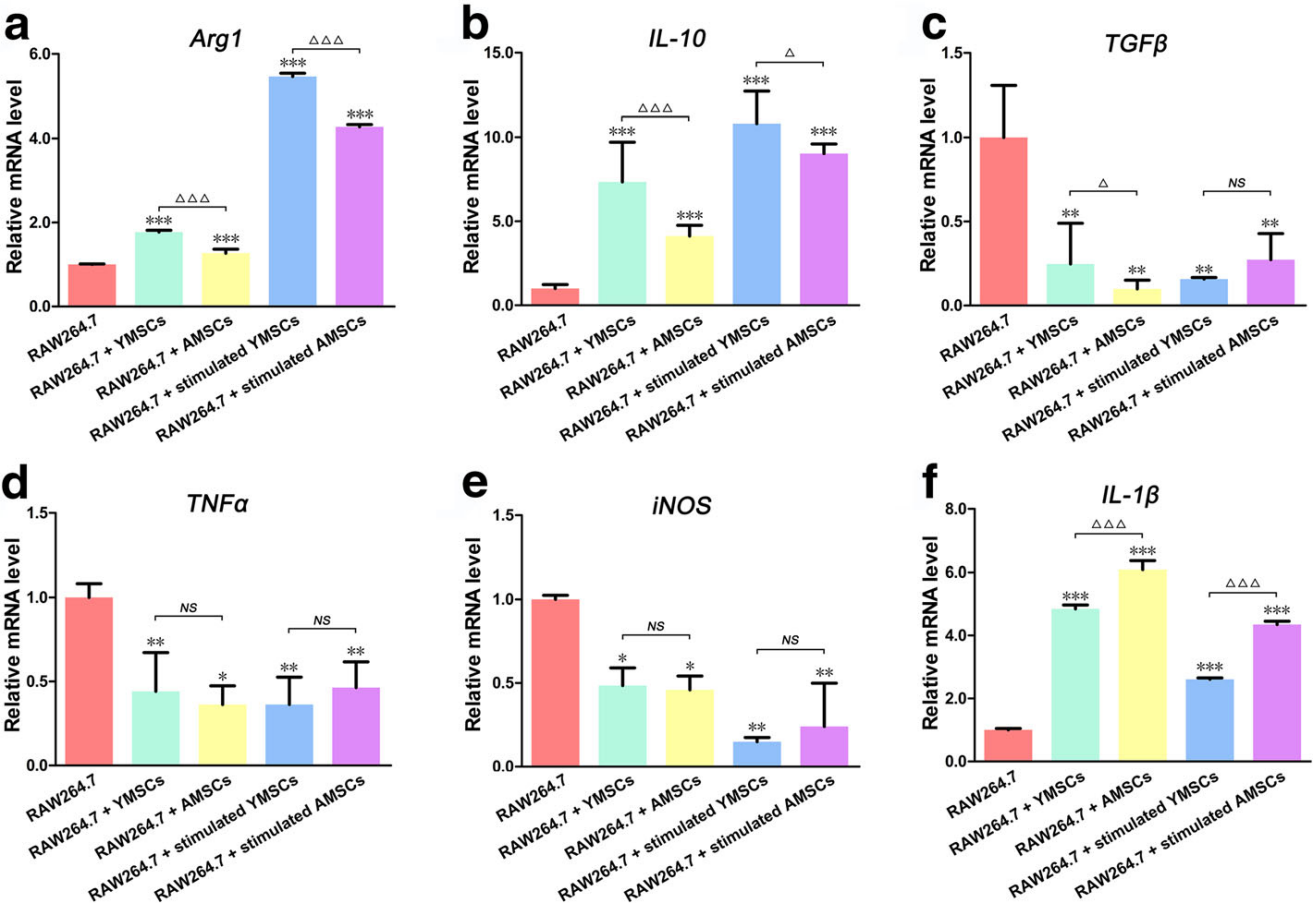
Gene expression levels of the M2-related enzyme/cytokines *Arg1* (**a**), *IL-10* (**b**) and *TGFβ* (**c**) as well as of the M1-related enzyme/cytokines *TNFα* (**d**), *iNOS* (**e**) and *IL-1β* (**f**), analyzed by qRT-PCR in RAW264.7 cells following coculture with YMSCs and AMSCs (with or without IFNγ stimulation); RAW264.7 cells without coculture served as control. Values are relative to *GAPDH* and normalized to the control group. Data shown as mean ± standard deviation. *p < 0.05, **p < 0.01, ***p < 0.001, significant differences when compared to control; ^△^ *p* < 0.05, ^△△△^ *p* < 0.001, a significant difference between the indicated groups; ^*NS*^ *p* > 0.05, no significant difference between the indicated groups. *AMSC* aged bone marrow-derived mesenchymal stem cell, *YMSC* young bone marrow-derived mesenchymal stem cell, *Arg1* Arginase-1, *IL* interleukin, *TGFβ* transforming growth factor beta, *TNFα* tumor necrosis factor alpha, *iNOS* inducible nitric oxide synthase

In terms of the qRT-PCR assay, the mRNA expression levels of an M2-related enzyme (*Arg1*, Fig. 4a) and cytokine (*IL-10*, Fig. 4b) in RAW264.7 Mφs were increased following coculture with BMSCs in all of the designed groups, while levels of an M1-related cytokine (*TNFα*, Fig. 4d) and enzyme (*iNOS*, Fig. 4e) were decreased. Although there were no significant differences between the expression levels of *TNFα* and *iNOS* in RAW264.7 cells following coculture with YMSCs and AMSCs (*p* > 0.05), coculture with YMSCs led to a more significant increase in *Arg1* and *IL-10* expression levels, irrespective of stimulation (*p* < 0.001 or *p* < 0.05). In contrast, levels of an M2-related cytokine (*TGFβ*, Fig. 4c) decreased while those of an M1-related cytokine (*IL-1β*, Fig. 4f) increased following coculture with BMSCs. When mRNA expression levels in RAW264.7 Mφs following coculture with YMSCs and AMSCs were compared, more *TGFβ* was expressed in RAW264.7 cells following coculture with unstimulated YMSCs (*p* < 0.05, Fig. 4c), while less *IL-1β* was expressed in RAW264.7 cells following coculture with YMSCs (with or without stimulation) (*p* < 0.001, Fig. 4f).

### IL-10, TNFα and NO production in supernatants of RAW264.7 cell coculture groups

**Fig. 5:**
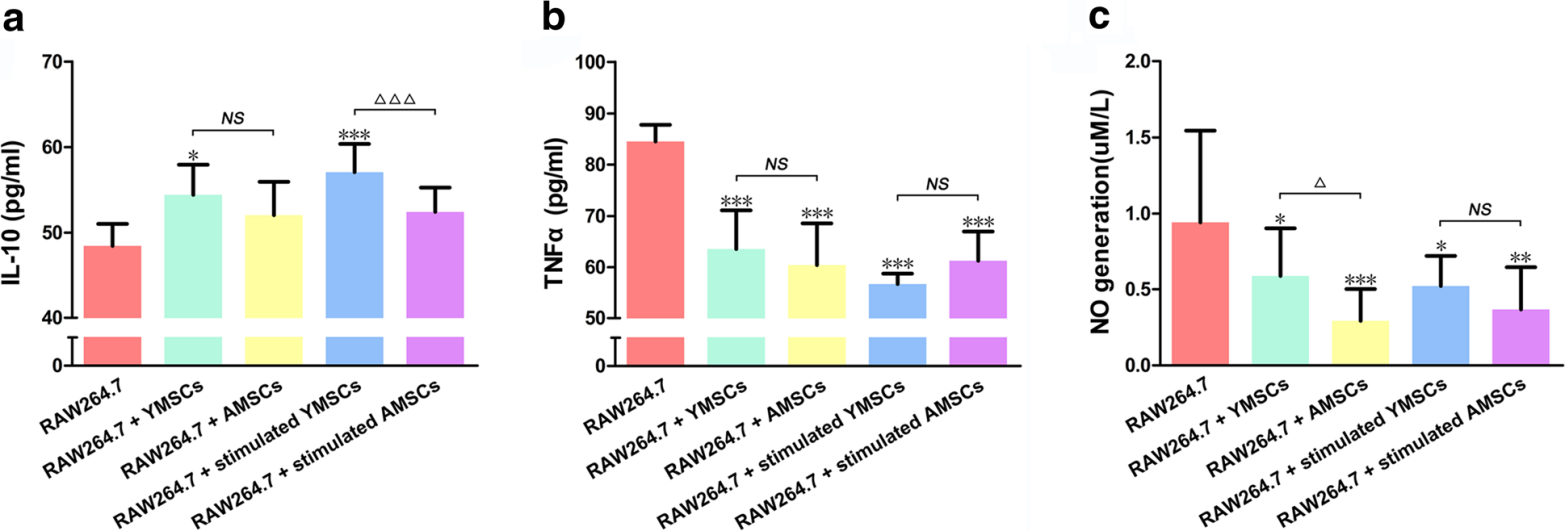
Generation of IL-10 (**a**), TNFα (**b**) and NO (**c**) in supernatants of RAW264.7 cell coculture groups; supernatants from RAW264.7 cells without coculture served as control. Values shown as mean ± standard deviation. *p < 0.05, **p < 0.01, ***p < 0.001, significant differences when compared to control; ^△^ *p* < 0.05, ^△△△^ *p* < 0.001, a significant difference between the indicated groups; ^*NS*^ *p* > 0.05, no significant difference between the indicated groups. *AMSC* aged bone marrow-derived mesenchymal stem cell, *YMSC* young bone marrow-derived mesenchymal stem cell, *IL-10* interleukin-10, *TNFα* tumor necrosis factor alpha, *NO* nitric oxide

Coculture with YMSCs (with or without IFNγ stimulation) increased IL-10 production in supernatants of RAW264.7 cell coculture groups (*p* < 0.001 or *p* < 0.05) (Fig. 5a), while all BMSC coculture treatments decreased TNFα and NO production (*p* < 0.001, *p* < 0.01, or *p* < 0.05) (Fig. 5b, c). Although coculture with stimulated YMSCs resulted in more IL-10 production (*p* < 0.05) (Fig. 5a) and coculture with unstimulated YMSCs resulted in more NO production (*p* < 0.05) (Fig. 5c), there were no significant differences in terms of IL-10, TNFα and NO production in supernatants between other matched coculture groups.

### Phagocytic assay of RAW264.7 cells following coculture

**Fig. 6:**
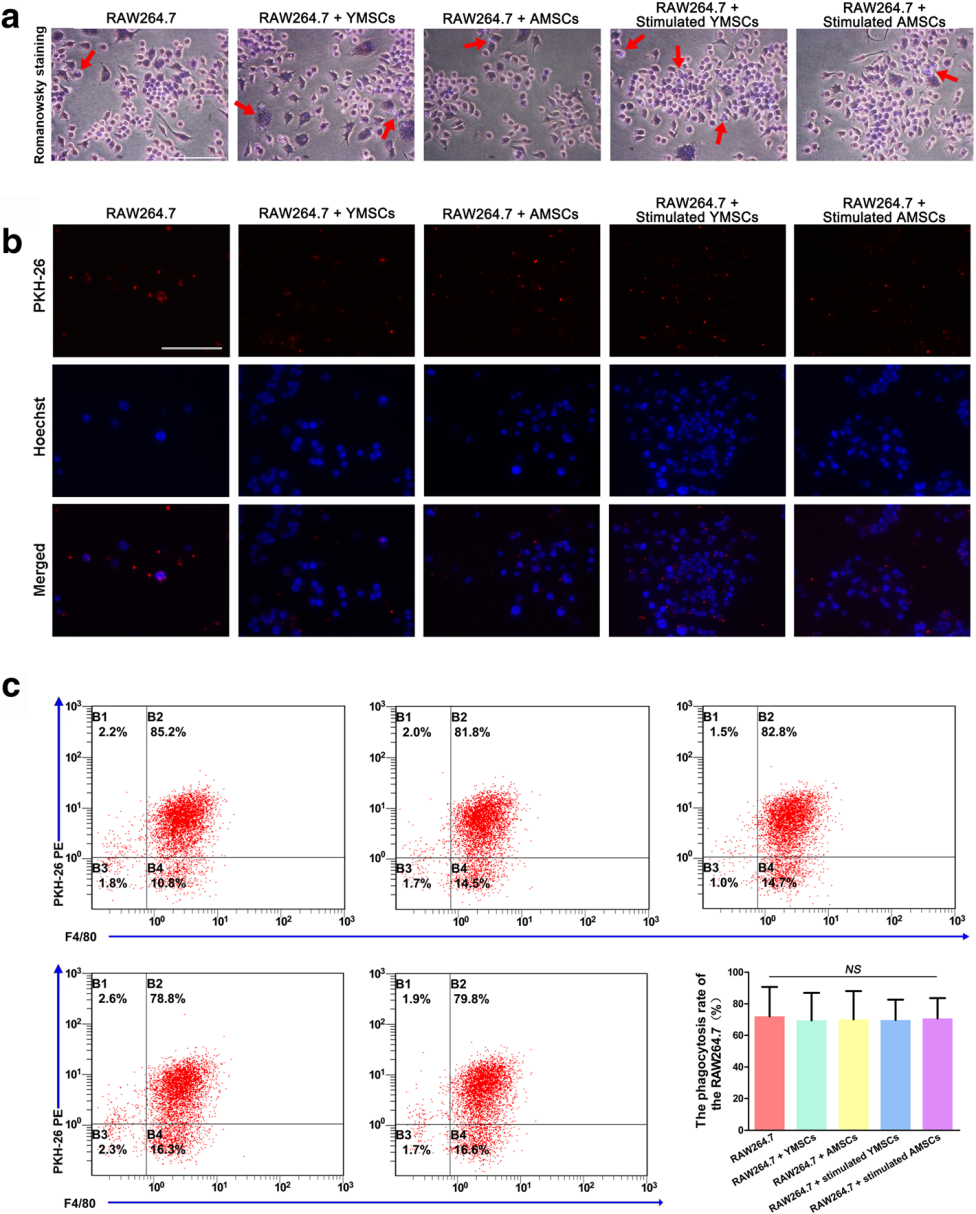
Phagocytosis of CRBCs by RAW264.7 cells following coculture with YMSCs and AMSCs (with or without IFNγ stimulation); RAW264.7 cells without coculture served as control. **a** Romanowsky staining for RAW264.7 cells after being incubated with CRBCs (*red arrows*, CRBCs being phagocytosed by RAW264.7 cells). **b** Fluorescence staining of RAW264.7 cells after being incubated with CRBCs; CRBCs were labeled with PKH-26 before being phagocytosed, and RAW264.7 cells were labeled by Hoechst following phagocytosis (*scale bar*: 100 μm). **c** Flow cytometric analysis of RAW264.7 cells (stained by FITC-F4/80 following phagocytosis) and CRBCs (labeled by PKH-26 before incubation). Data shown as mean ± standard deviation. ^*NS*^ *p* > 0.05, no significant difference between the indicated groups. *AMSC* aged bone marrow-derived mesenchymal stem cell, *YMSC* young bone marrow-derived mesenchymal stem cell (Color figure online)

With respect to Romanowsky staining, CRBCs presented as small round light cells, while RAW264.7 cells were stained with a blue nucleus and pink cytoplasm. RAW264.7 cells exhibited obvious phagocytosis when they were incubated with CRBCs (Fig. 6a). Fluorescence staining demonstrated that PKH-26-labeled CRBCs (red) that were closely adjacent to blue nuclei had been phagocytosed by RAW264.7 cells (Fig. 6b). Phagocytosis was further confirmed by flow cytometry, but coculture of RAW264.7 cells with either YMSCs or AMSCs (with or without IFNγ stimulation) had no significant influence on phagocytosis (Fig. 6c).

### Migration ability of RAW264.7 cells following coculture

**Fig. 7:**
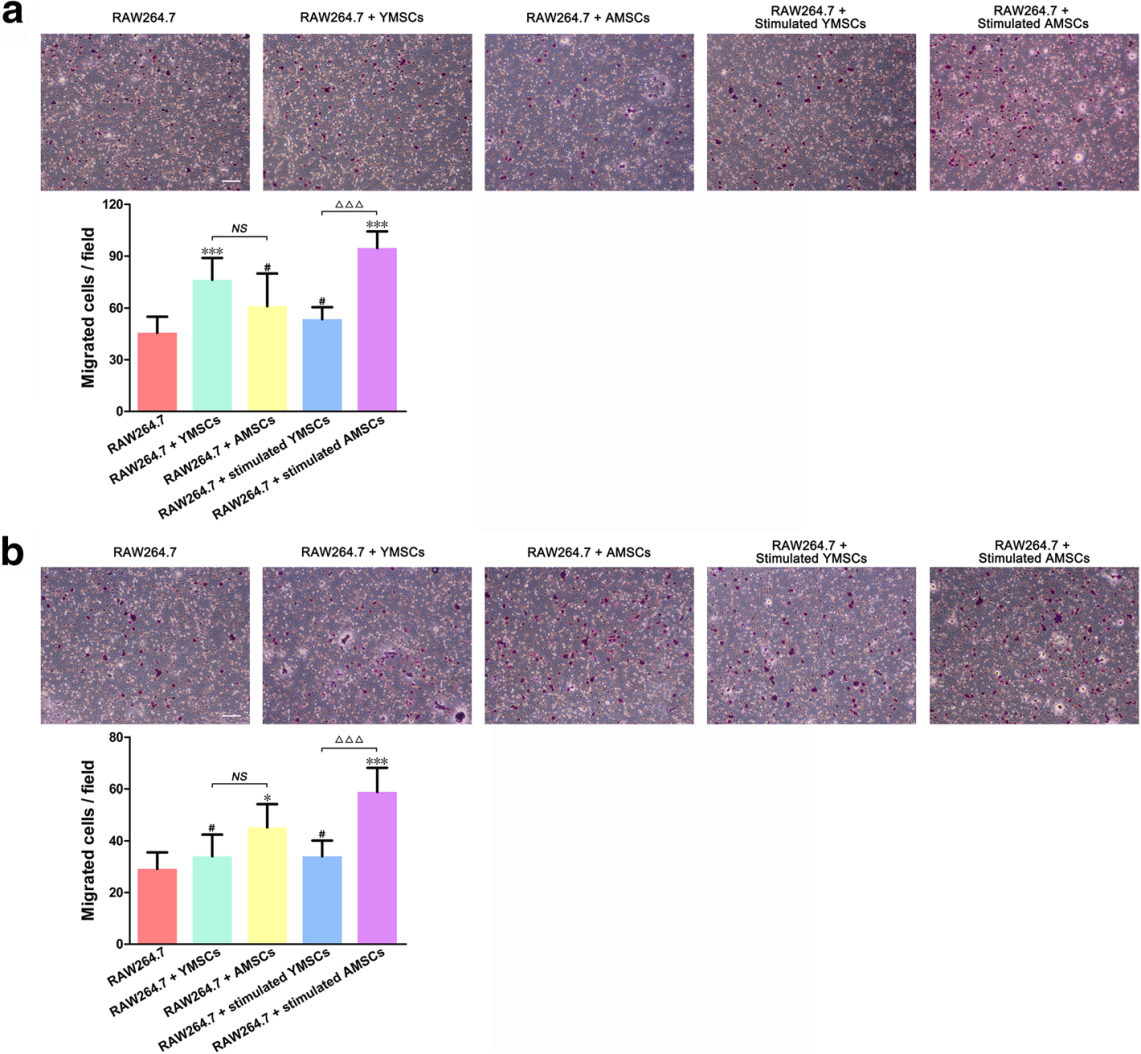
Cell migration of RAW264.7 cells in Transwell systems during (**a**) or following (**b**) coculture with YMSCs and AMSCs (with or without IFNγ stimulation); RAW264.7 cells without coculture served as control. **a** BMSC chemotaxis migration assay: RAW264.7 cells were placed in the inserts (upper compartments), while BMSCs were placed in the plates (lower compartments); representative images (*scale bar*: 100 μm) were taken 10 h after transporting RAW264.7 cells to the Transwell systems (BMSCs were preattached in the lower plate), and statistical analysis was performed by counting the number of migrated cells in different groups. **b** Mφ migration assay: RAW264.7 cells following coculture (without coculture as control) were placed in the inserts (upper compartments), while no cells were placed in the plates (lower compartments); representative images (*scale bar*: 100 μm) were taken 10 h after transporting RAW264.7 cells to the Transwell system, and statistical analysis was performed by counting the number of migrated cells in different groups. *p < 0.05, ***p < 0.001, a significant difference in the number of migrated cells compared to control; ^△△△^ *p* < 0.001, a significant difference between the indicated groups; ^*NS*^ *p* > 0.05, no significant difference between the indicated groups. *AMSC* aged bone marrow-derived mesenchymal stem cell, *YMSC* young bone marrow-derived mesenchymal stem cell

In a BMSC chemotaxis migration assay, more RAW264.7 cells were recruited by stimulated AMSCs than by stimulated YMSCs (*p* < 0.001), but such differences were not observed in RAW264.7 cells recruited by unstimulated YMSCs and AMSCs (*p* > 0.05) (Fig. 7a). When the migration ability of RAW264.7 cells following coculture with BMSCs was tested in an Mφ migration assay, more RAW264.7 Mφs migrated across the inserts following coculture with stimulated AMSCs than those cocultured with stimulated YMSCs (*p* < 0.001). Again, coculture with YMSCs and AMSCs resulted in no differences in the number of migrated RAW264.7 cells (*p* > 0.05) (Fig. 7b).

## Discussion

MSCs are a cell population that contains precursors for multiple mesenchymal cell lineages and can differentiate into various tissue-specific cells, such as osteoblasts, chondrocytes and adipocytes [36, 37]. In the past decade, our understanding of MSC-based regeneration and its underlying mechanisms has continued to expand markedly, and recent advances in three-dimensional culture systems as well as in-situ culture counterparts have led to fresh insights into cell physiology and the role of MSCs in the immune system [16]. There is a growing body of evidence which shows that MSCs can affect immunomodulatory functions, typically by regulating the function of immune cells [32, 38, 39]. Previous investigations have found significant changes among MSCs obtained from donors of different ages, including but not limited to differences in proliferation and gene expression (reviewed in [40, 41]), and cytokines in the local environment can affect their immunomodulatory function [32–34]. Therefore, we investigated how BMSCs of young and aged mice affect the phenotype and functions of Mφs using in-vitro cell systems. Referencing the reported data, we also used IFNγ at a concentration of 20 ng/ml to stimulate BMSCs to investigate whether cell pretreatment by cytokines can impact cell immunomodulation.

Although MSCs have already been applied to modulate Mφs and their downstream functions [23, 26], we used RAW264.7 cells as the source of Mφs in the present study, which not only eliminated the instability and immaturity of mouse-derived Mφs in vivo but also avoided possible influences from other cells mixed with in-vivo isolated Mφs. The characteristics of RAW264.7 Mφs following coculture with YMSCs and AMSCs, with or without IFNγ stimulation, were evaluated and contrasted, wherein phenotype-related markers of Mφs were determined using flow cytometry and immunofluorescence, and qRT-PCR and ELISA assays were used to clarify phenotype-related gene expression and cytokine or NO production. Finally, we compared the phagocytic and migratory capacities of RAW264.7 cells in response to coculture with BMSCs in different groups.

In accordance with previous reports [1, 2, 42], YMSCs displayed a higher proliferative capacity and a greater osteogenic potential than AMSCs, which were more likely to undergo adipogenic differentiation (Fig. 1). To determine the effect of aging on the immunomodulatory properties of MSCs, we applied YMSCs and AMSCs to coculture with RAW264.7 cells in Transwell systems, where RAW264.7 cells without coculture served as the control. In flow cytometry analysis, cell markers (CD11b and F4/80) demonstrated the purity of Mφs (Fig. 2a). Following coculture, the surface marker CD86 was significantly decreased, whereas CD206 was greatly increased (Fig. 2b, c). Coculture with BMSCs can also modify the shape of Mφs. Compared to the control, RAW264.7 cells are relatively small, round and regular (Fig. 3a). Without stimulation, YMSCs and AMSCs led to similar CD86 and CD206 profiles in cocultured Mφs (*p* > 0.05); however, stimulated YMSCs exhibited an enhanced ability to coax M2 polarization, as shown by the higher CD206 expression in Mφs cocultured with YMSCs following stimulation (Fig. 2c). When the expression of iNOS and CD206 in RAW264.7 cells was further observed using an immunofluorescence assay, coculture with either YMSCs or AMSCs, with or without stimulation, consistently decreased Mφ iNOS expression and at the same time promoted CD206 expression in RAW264.7 cells (Fig. 3b). Given that CD86 and iNOS are mostly expressed in the proinflammatory M1 phenotype of Mφs [43, 44], while CD206 is prominent in M2 phenotype cells [45, 46], we conclude that coculture with BMSCs (particularly those that have undergone stimulation) can induce Mφs toward M2 polarization; these findings are generally in line with data reported previously [22–27].

Mφs actively infiltrate into wound areas, and by secreting different cytokines and enzymes they participate in combating microbes and relate innate immunity to adapted immunity [18, 19]. Therefore, gene expression of key cytokines and enzymes in cells as well as cell capacity for molecular production are important parameters to evaluate Mφ function. Similar to previous findings [24, 26, 47], our data also showed that BMSCs were able to restrain proinflammatory cytokine (TNFα) and M1-related marker (iNOS) expression as well as promote anti-inflammatory cytokine (IL-10) and M2-related marker (Arg1) expression in RAW264.7 cells (Figs. 4 and 5). Further, we found that YMSCs, with or without stimulation, exhibited an enhanced potential for modulating RAW264.7 expression of anti-inflammatory cytokines (IL-10) and M2-related markers (Arg1) when compared to AMSCs. Although TGFβ contributes to immune suppression and inflammation modulation [48], coculture with BMSCs decreased TGFβ expression in RAW264.7 cells in the present study (Fig. 4c). In fact, it is still controversial whether MSC treatment contributes to an increase or a decrease in TGFβ expression in Mφs [49, 50]. Similarly, expression of the proinflammatory cytokine IL-1β in RAW264.7 cells was increased after coculture with BMSCs (Fig. 4f). These data are not consistent with literature reports published previously in which MSC treatment was found to decrease the levels of IL-1β in LPS-activated or other inflammasome-activated Mφs [51, 52]. However, RAW264.7 cells were not exposed to proinflammatory stimuli prior to coculture with BMSCs. Furthermore, there is evidence that without the paracrine loop of IL-1β signaling, immune suppression of MSCs will be weakened [51]. These reports may partially explain the increase in IL-1β in MSC-treated Mφs.

Derived from Mφs (mostly M1) or other immune cells, NO plays a large role in combating pathogens [44, 53]. We found that coculture with BMSCs decreased NO generation in Mφs (Fig. 5c), which was in agreement with what has been reported previously [54]. Interestingly, without stimulation, AMSC coculture suppressed NO production in RAW264.7 cells more effectively than YMSC coculture. The difference in capacity for NO production by RAW264.7 cells might derive from more NO being produced by aged MSCs than by young MSCs, and NO was found to take part in suppressing M1 subtype differentiation in vivo [55, 56].

Phagocytosis is one of the important characteristics of Mφs, which can function as scavenger cells (phagocytize cell debris and pathogens) and link innate immunity and adaptive immunity through antigen presentation [57] and are closely related to the transformation from proinflammatory M1 phenotypes toward anti-inflammatory M2 phenotypes [30]. Data from the phagocytic assay demonstrated that there were no significant differences in phagocytosis among RAW264.7 cells in different treatment groups (Fig. 6); this finding is not completely consistent with previous reports [23, 27, 58]. In fact, phagocytosis is not simply related to M1 or M2 phenotype. An in-vitro study determined that apart from M1, an M2a phenotype is induced by IL-4/IL-13, M2c is induced by IL-10/TGFβ [59], and IL-10-activated Mφs contribute to efficient clearance of apoptotic cells [60, 61]. Hence, age-related changes in phagocytosis in RAW264.7 cells and the associated mechanisms are worthy of further investigation.

Although mounting evidence has demonstrated the multifunctional roles of Mφs [4, 62], effective participation of Mφs in tissue repair depends on their ability to be recruited to the site of action. Hence, we designed a Transwell system to study the migration of RAW264.7 cells in response to coculture with various BMSCs. When RAW264.7 cells were placed in the inserts (upper compartments) while BMSCs were placed in the plates (lower compartments), RAW264.7 cells exhibited an enhanced migratory potential (Fig. 7a). Without stimulation YMSCs induced more migration in Mφs, while following stimulation AMSCs were more potent in coaxing Mφ migration; these findings were partially consistent with other studies [26, 63, 64]. When RAW264.7 cells following coculture were placed in the inserts (upper compartments) without BMSCs in the plates (lower compartments), coculture with AMSCs resulted in more migratory Mφs compared to coculture with YMSCs, and a significant difference was found following RAW264.7 cell coculture with stimulated YMSCs and stimulated AMSCs (Fig. 7b). As indicated previously, Mφs can be recruited by a large number of chemokines, for instance monocyte chemoattractant protein 1 (MCP-1) and macrophage inflammatory protein 1 alpha [63–65]—in fact the MCP-1 expression level in aged MSCs is relatively increased [66]. This can explain why aged BMSCs recruited more Mφs. Recently, Li et al. [67] found that IFNγ combined with TNFα promotes MCP-1 production in human umbilical cord MSCs, suggesting that stimulation with cytokines can enhance the ability of MSCs to recruit Mφs, but this enhancement was only found in stimulated AMSCs in the present study (Fig. 7a). Similarly, in an Mφ migration assay following coculture, stimulated AMSCs promoted the migratory capacity of RAW264.7 cells more significantly (Fig. 7b). Overall, we found that stimulated AMSCs performed better in recruiting RAW264.7 cells and in promoting the migration capacity of RAW264.7 cells through coculture.

Complex cell–cell interactions between MSCs and Mφs within a local niche play crucial roles in regulating wound healing and regeneration. Because of their regulatory capacities, MSCs exert a functional role in modulating Mφ polarization and hence the downstream immune responses that are beneficial for tissue repair [28–31, 68]. On the other hand, Mφ subsets and particularly their secretome can influence the therapeutic potentials of MSCs [62, 69]. In fact, complex crosstalk exists via which MSCs and Mφs communicate, creating a feedback loop which contributes to the overall immunomodulatory function identified following stem cell therapy. Both the immune modulatory and regenerative potentials of MSCs are affected by increasing age, which is potentially associated with lower baseline levels of anti-inflammatory and immunomodulatory mediators, and a significant reduction in their proliferative and tissue-specific differentiation capabilities [70]. Much remains to be done before aging cells can be used in a clinical setting for cytotherapy and regenerative medicine.

## Conclusion

In our research, we demonstrated that both YMSCs and AMSCs possess the ability to modulate the phenotype and functions of Mφs; wherein YMSCs exhibited a greater potential to steer M2 polarization and IFNγ stimulation was able to modify the MSC influence on Mφs, but an optimized stimulation to enhance cell immunomodulation of BMSCs remains to be identified. Based on these findings, we conclude that aging MSCs experience a decline in stem cell function and regenerative potential, which can at least in part be attributed to their impaired immunomodulatory properties. Understanding the mechanism underlying this age-associated deterioration of MSC function and identification of effective strategies to rescue aging stem cells are of equal importance for developing stem cell-based therapies.
